# COVID-19 vaccination in pregnancy: views and vaccination uptake rates in pregnancy, a mixed methods analysis from SAIL and the Born-In-Wales Birth Cohort

**DOI:** 10.1186/s12879-022-07856-8

**Published:** 2022-12-12

**Authors:** Mohamed Mhereeg, Hope Jones, Jonathan Kennedy, Mike Seaborne, Michael Parker, Natasha Kennedy, Sarah Beeson, Ashley Akbari, Luisa Zuccolo, Alisha Davies, Sinead Brophy

**Affiliations:** 1 National Centre for Population Health and Wellbeing Research, Faculty of Medicine, Health and Life Science, Swansea University Medical School, Swansea, Wales UK; 2 Population Data Science, Faculty of Medicine, Health and Life Science, Swansea University Medical School, Swansea, Wales UK; 3 Department of Population Health Sciences, Bristol Medical School, University of Bristol, Bristol, England UK; 4 Research and Evaluation Division, Public Health Wales, Swansea, UK

**Keywords:** COVID-19 vaccination, Pregnancy, Vaccine uptake, Vaccine hesitancy, SAIL

## Abstract

**Background:**

Vaccine hesitancy amongst pregnant women has been found to be a concern during past epidemics. This study aimed to (1) estimate COVID-19 vaccination rates among pregnant women in Wales and their association with age, ethnicity, and area of deprivation, using electronic health record (EHR) data linkage, and (2) explore pregnant women’s views on receiving the COVID-19 vaccine during pregnancy using data from a survey recruiting via social media (Facebook, Twitter), through midwives, and posters in hospitals (Born-In-Wales Cohort).

**Methods:**

This was a mixed-methods study utilising routinely collected linked data from the Secure Anonymised Information Linkage (SAIL) Databank (Objective 1) and the Born-In-Wales Birth Cohort participants (Objective 2). Pregnant women were identified from 13th April 2021 to 31st December 2021. Survival analysis was utilised to examine and compare the length of time to vaccination uptake in pregnancy, and variation in uptake by; age, ethnic group, and deprivation area was examined using hazard ratios (HR) from Cox regression. Survey respondents were women who had a baby during the COVID-19 pandemic or were pregnant between 1st November 2021 and 24th March 2022 and participating in Born-In-Wales. Codebook thematic analysis was used to generate themes from an open-ended question on the survey.

**Results:**

Population-level data linkage (objective 1): Within the population cohort, 8203 (32.7%) received at least one dose of the COVID-19 vaccine during pregnancy, 8572 (34.1%) remained unvaccinated throughout the follow-up period, and 8336 (33.2%) received the vaccine postpartum. Younger women (< 30 years) were less likely to have the vaccine, and those living in areas of high deprivation were also less likely to have the vaccine (HR = 0.88, 95% CI 0.82 to 0.95). Asian and Other ethnic groups were 1.12 and 1.18 times more likely to have the vaccine in pregnancy compared with White women (HR = 1.12, 95% CI 1.00 to 1.25) and (HR = 1.18, 95% CI 1.03 to 1.37) respectively. Survey responses (objective 2): 207 (69%) of participants stated that they would be happy to have the vaccine during pregnancy. The remaining 94 (31%) indicated they would not have the vaccine during pregnancy. Reasons for having the vaccine included protecting self and baby, perceived risk level, and receipt of sufficient evidence and advice. Reasons for vaccine refusal included lack of research about long-term outcomes for the baby, anxiety about vaccines, inconsistent advice/information, and preference to wait until after the pregnancy.

**Conclusion:**

Potentially only 1 in 3 pregnant women would have the COVID-19 vaccine during pregnancy, even though 2 in 3 reported they would have the vaccination, thus it is critical to develop tailored strategies to increase its acceptance rate and decrease vaccine hesitancy. A targeted approach to vaccinations may be required for groups such as younger people and those living in higher deprivation areas.

**Supplementary Information:**

The online version contains supplementary material available at 10.1186/s12879-022-07856-8.

## Background

Vaccination is acknowledged as a successful public health measure [1]. However, a growing number of the general population perceive vaccinations as unsafe and nonessential [1]. The Scientific Advisory Group for Emergencies (SAGE) working group described vaccine hesitancy using a ‘3 C’s’ model; Confidence, Complacency, and Convenience [2]. The model suggests that vaccine hesitancy arises when individuals (a) do not have confidence in the vaccine's safety and effectiveness, (b) do not believe in the seriousness of the disease and, (c) have the perception that access to the vaccine is inconvenient. Combatting the 3 C’s may lead to higher vaccine acceptance.

Vaccine hesitancy may be more common in pregnant women [3]. During the COVID-19 pandemic, the limited data and changes in advice/recommendations regarding the COVID-19 vaccination in pregnancy led to some hesitancy among pregnant women in certain settings [4]. Misleading information on vaccine safety spread on social media platforms linking the COVID-19 vaccine to infertility [4]. The lack of long-term safety data in pregnancy on the fetus or child reportedly lead to higher levels of distrust, and apprehension regarding the vaccine safety among pregnant women or those trying to conceive, as shown by studies from America [4].

Low vaccine uptake among pregnant women carries implications for both clinical and population health outcomes. Unvaccinated pregnant women are at increased risk of requiring hospital treatment for COVID-19 compared to those who are vaccinated [5]. Severe COVID-19 in pregnancy significantly increases the risks to the baby [6]. Pregnant women with severe COVID-19 were more likely to have a preterm birth, have a pre-labour caesarean birth, have a baby that was stillborn or be admitted to a neonatal intensive care unit [6].

In the UK, the COVID-19 vaccination programme started on 8th December 2020, prioritising individuals at greater risk of being hospitalised or contracting severe cases of COVID-19 and individuals who care for vulnerable groups, such as health and social care workers. At this time, the UK’s Joint Committee on Vaccination and Immunisation (JCVI) guidance was that the COVID-19 vaccine should not be given to pregnant women as there was a lack of data regarding the safety of the COVID-19 vaccine during pregnancy. Later, in April 2021, the UK JCVI announced that pregnant women should be offered the COVID-19 vaccine [7].

Research is limited on population-level COVID-19 vaccine uptake in pregnancy in the UK. In Scotland, a national, prospective cohort study identifying ongoing pregnancies through extensive electronic health record (EHR) data linkage showed vaccination rates in pregnant women to be substantially lower than in the general population; 32.3% in pregnant women compared to 77.4% in all women [8]. In England, 22.7% of women giving birth in August 2021 had received at least one dose of vaccine. This increased to 32.3% of women who gave birth in September—and the latest data shows that it rose to 53.7 in December 2021. As of 30th June 2021, more than 70% of the Welsh population has received a first dose of a COVID-19 vaccine, with 53% of the population receiving second doses [9]. Despite the overall increase in coverage, the uptake remains lower amongst pregnant women compared to the general population of the same age group [5, 10].

In research aimed to determine the attitudes toward vaccine acceptance and hesitancy of the COVID-19 vaccine in pregnant women [11], it was observed that 37% of pregnant women stated they intended to receive the vaccine if it was recommended for pregnant women. The most common reasons stated for refusing the vaccine included lack of data about COVID-19 vaccine safety in pregnant populations and potential harm to the fetus. Identifying attitudes towards the COVID-19 vaccine among pregnant women will be beneficial for generating vaccination strategies that increase uptake during the pandemic.

The acceptance of the COVID-19 vaccine among pregnant women and mothers of young children was investigated in 16 countries worldwide [12]. The strongest predictors of vaccine acceptance included confidence in vaccine safety or effectiveness, worrying about COVID-19, belief in the importance of vaccines to their own country, trust of public health agencies/health science, as well as attitudes towards routine vaccines [12].

While several studies have investigated vaccine hesitancy during pregnancy [3, 4], studies conducted in Wales during the COVID-19 pandemic are lacking. In addition, there are a lack of studies examining reasons for COVID-19 vaccine hesitancy during pregnancy in combination with national-level data on actual vaccination uptake for this population.

The aims of this study are to Objective (1a) use national health data linkage of COVID-19 vaccination and pregnancy records to identify vaccine uptake amongst pregnant women in Wales, Objective (1b) examine differences by age, ethnic group, area of deprivation and Objective (2a) gain an insight into views and opinions on COVID-19 vaccine during pregnancy in a cross-section of pregnant women in Wales.

## Methods

### Study design and setting

A cohort study utilising routinely collected anonymised population-scale, individual-level linked data from the Secure Anonymised Information Linkage (SAIL) Databank. Data sources include general practitioners (GP), hospital admissions, national community child health, maternal indicators, and vaccination data sources. All women recorded as being pregnant between the 13th April 2021 and 31st December 2021, aged 18 years or older, and eligible for COVID-19 vaccination were identified. They were linked to the COVID-19 vaccination data for dates from 7th December 2020 up to and including 31st December 2021.

Pregnant women in Wales were invited through the Born-In-Wales study to complete an online survey via social media (Facebook and Twitter), recruitment through midwives, and posters in hospitals. Respondents participating in Born-In-Wales were women who had a baby during the pandemic or who were currently pregnant when the questionnaire was live from the 1^st^ November 2021 to 24th March 2022. The main open-ended questions employed were ‘what is your view on having the COVID-19 vaccine in pregnancy?’, and ‘have you had, or would you have, the COVID-19 vaccine while pregnant and why?’. All responses were anonymous, and the self-assessed inclusion criteria were living in Wales and either being pregnant or having had a baby during the COVID-19 pandemic.

### Data sources and linkage

Analysis was undertaken using anonymised population-scale, individual-level linked routinely collected national-scale data available in the SAIL Databank [13, 14], which anonymously links a wide range of person-based data using a unique personal identifier. The linkage is brought together under the Born-In-Wales study [15] and includes Wales Longitudinal General Practice (WLGP) records linked with hospital admission inpatient from Patient Episode Database for Wales (PEDW) and outpatient from Outpatient Database for Wales (OPDW)) records, the National Community Child Health (NCCH), Maternal Indicators (MIDS) and the COVID-19 Vaccination (CVVD) data. The WLGP system utilises Read codes, which are 5-digit codes that relate to diagnosis, medication, and process of care codes. The secondary care data uses ICD-10 codes for diagnosis and surgical interventions. The NCCH comprises information about birth registration, child health examination monitoring, and immunisations. The MIDS data contains data relating to the woman at initial assessment and mother and baby (or babies) for all births. In addition to these data sources, the Welsh Demographic Service Dataset (WDSD) was linked to extract Lower-layer Super Output Area (LSOA) version 2011 information associated with area level deprivation. In particular, the Welsh Index for Multiple Deprivation (WIMD) 2019 was employed as a proxy to assess social deprivation. These records were linked at the individual-level for all women known to be pregnant in Wales between 13th April 2021 and 31st December 2021 and then stratified for subanalysis by age group, ethnic group, and WIMD quintile. Linkage quality has been assessed and reported as 99.9% for WLGP records and 99.3% for PEDW records [16]. All linkage was at the individual level.

### Study population and key dates

Pregnant women were identified as any woman with pregnancy codes in the WLGP or PEDW data, or mothers in the NCCH or MIDS data with the baby's birth date (pregnancy end date) and gestational age at birth available. The baby’s birth date and gestational age enabled the start date of pregnancy to be determined for those who gave birth during the study period. Data collected included vaccination data, Welsh index of multiple deprivation (WIMD 2019), and ethnic group. The WIMD is an official measure for the relative deprivation of areas of Wales. It combines eight separate domains of deprivation, each compiled from a range of different indicators (income, employment, health, education, access to services, housing, community safety, and physical environment), into a single score and is widely used to measure deprivation in Wales. Ethnic group was categorised in SAIL into White, Mixed, Asian, Black, and Other.

The study start date of 13th April 2021 was selected because phase 2 of the vaccination program, which aimed to provide vaccinations to individuals aged 40 to 49, 30 to 39, and 18 to 29 years, commenced on this date. The inclusion criteria were currently pregnant women who had not received the vaccination or had one dose of vaccination before pregnancy, alive, known pregnant on the first day of follow-up, and aged 18 years or older. The exclusion criteria were women who were fully vaccinated (i.e. two vaccinations) before pregnancy, those for whom it was not possible to determine the start date of pregnancy due to unavailability of the gestational age and initial assessment dates in their records or those with miscarriage or stillbirth outcomes.

### Calculating pregnancy start date

Pregnancy start dates were calculated from the following sources:

For pregnancies identified from the NCCH and MIDS data, the pregnancy start dates were calculated based on the gestational age and the week of birth data items available in these data sources. In cases where gestational age is missing, a value of 40 weeks was used as the majority with missing data (92%) had birth weights suggestive of full-term infants. Thus, the pregnancy start date (last menstrual period) was simply calculated by subtracting the gestational age at birth (in weeks) from the week of birth. Pregnancies identified from both data sources were compared/matched, and duplicate records were removed.

For pregnancies identified from the WLGP data, all pregnant women with a pregnancy code and event date that occurred during the study period were extracted (Additional file 1: Table S1). For those identified from the hospital admissions data (PEDW), all women with a pregnancy diagnosis code and an attendance date occurring during the study period were also extracted (Additional file 1: Table S2). Identified cases from both the WLGP and PEDW were separately matched to those identified from the NCCH and MIDS data to include only those who are still pregnant. Furthermore, the identified cases from both resources were further matched to remove duplicates, then linked to the initial assessment-related data items in the MIDS data. The gestational age in weeks and initial assessment data items are available in order to calculate the pregnancy start date. In cases where multiple records were found per pregnant woman, only the first occurring record between the study dates of interest was selected. The pregnancy start date for every successfully linked case was then calculated by subtracting the gestational age from the initial assessment date.

### Survey methods

Pregnant and postpartum women during the COVID-19 pandemic were invited to complete an online survey via social media advertising. Codebook thematic analysis [17] was used to generate themes from an open-ended question on the survey: ‘What is your view on having the COVID vaccination in pregnancy, have you or would you have the COVID vaccination when pregnant and why?’. Thematic analysis identifies and describes patterns across data [17]. Analysis involved six phases (1) data familiarisation and writing familiarisation notes (2) systematic data coding (3) generating initial themes from coded and collated data (4) developing and reviewing themes (5) refining, defining, and naming themes and (6) writing the report. All data were independently analysed by HJ and SB, who then discussed their findings. This was to ensure that important concepts within the data were not missed, and to achieve a richer understanding of the data through multiple perspectives.

### Statistical analysis

Descriptive statistics were conducted on rates of vaccination uptake per month during pregnancy among women eligible for vaccination, stratified by age group. We further stratified ethnic group and area of deprivation uptake rates by age group. Kaplan–Meier survival analysis was employed to examine the time to vaccination and censored at birth, death, or moved out of Wales while pregnant. The log rank test was used to determine if there were differences in the survival distributions of vaccine uptake times within the different demographic variables. Differences were reported in median times (MD) with 95% confidence intervals and significance level accepted at p < 0.05. Multivariate Cox regression hazard models were utilised to examine the impact of the explanatory variables age group, ethnicity, and area of deprivation jointly on vaccination uptake, reporting hazard ratios (HR) with 95% confidence intervals and significance level accepted at p < 0.05. The reference groups were those aged 25–29, white ethnicity, and those living in the most affluent area. The data handling and preparation for the descriptive statistics, survival analysis and Cox proportional hazard modelling were performed using SQL on a IBM DB2 database within the SAIL Databank utilising Eclipse. Final data preparation specific to these analyses, such as setting the reference groups, was performed in IBM SPSS Statistics 28. Descriptive statistics were performed in Microsoft Excel 2016, and Survival/Cox regression analyses were performed in SPSS.

## Results

**Fig. 1:**
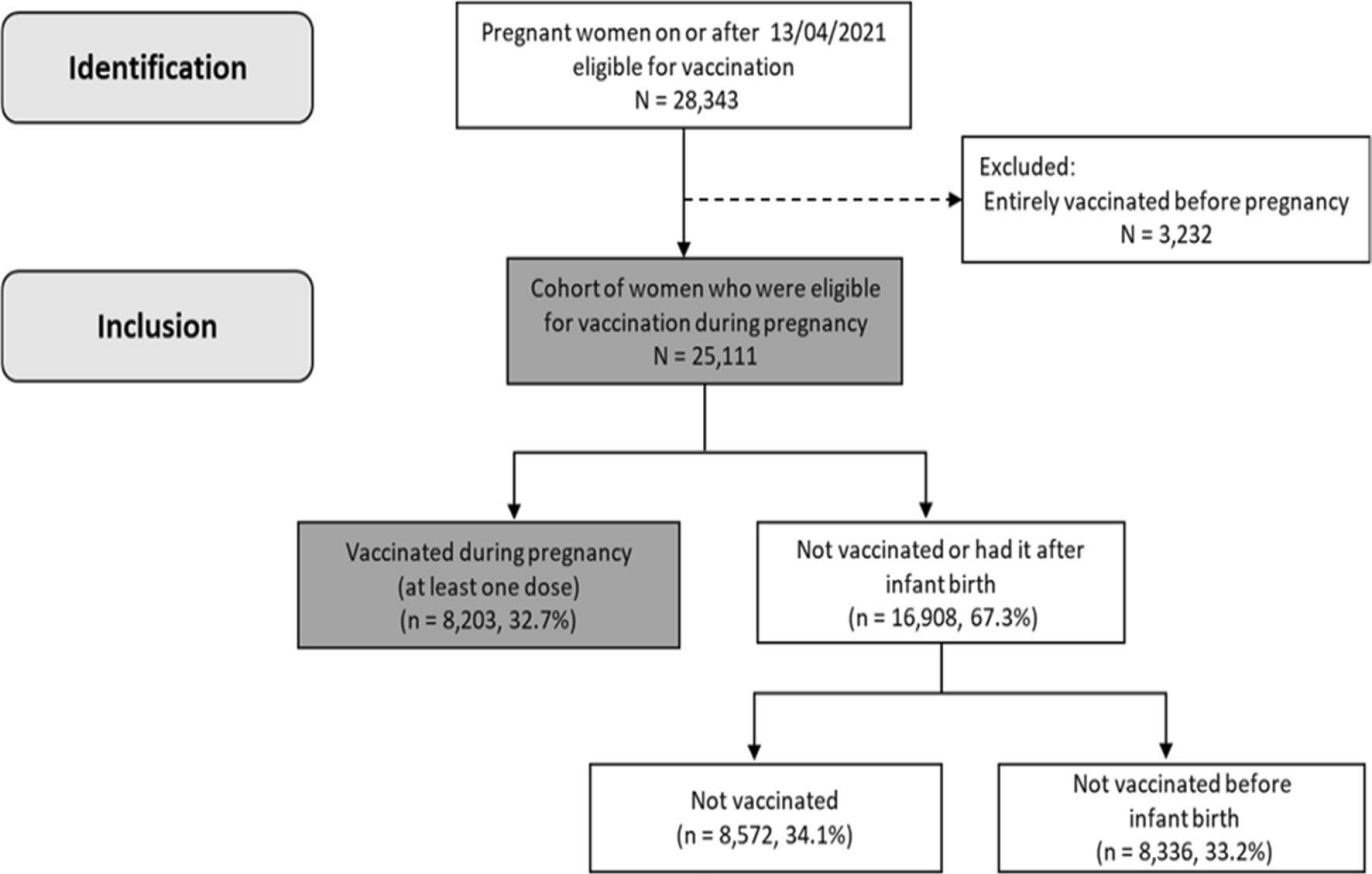
Flowchart of the cohort identification

A total of 28,343 pregnant women were identified from 13th April 2021 through 31st December 2021. After excluding women who were fully vaccinated before pregnancy (n = 3232), the cohort comprised of 25,111 pregnant women. Those women were followed up, and their records were linked to the COVID-19 vaccination data up to and including 31^st^ December 2021. (Fig. 1 describes the participants in the cohort). Most of the women were aged between 30 and 39, and between 25 and 29 years (48.4% and 29.7% respectively). The majority were White (77.8%). Nearly a quarter lived in the most deprived quintile (23.3%), and 14.4% were in the least deprived quintile (Table 1).

**Table 1:** Descriptive summaries of the pregnant women eligible for vaccination

		N	%
Age group	18–24	4664	18.6
	25–29	7447	29.7
	30–39	12,143	48.4
	40–50	857	3.4
Ethnic group	White^1^	19,547	77.8
	Asian^2^	902	3.6
	Other^3^	571	2.3
	Mixed^4^	316	1.3
	Black^5^	440	1.8
	Unknown	3335	13.3
WIMD Quintile 2019	1st (Most deprived)	5840	23.3
	2nd	4795	19.1
	3rd	4157	16.6
	4th	3804	15.1
	5th (Least deprived)	3626	14.4
	Unknown	2889	11.5

^1^Comprises of Any White Background, Gypsy or Irish Traveller

^2^Comprises of Bangladeshi, Pakistani, Indian, Any Other Asian Background

^3^Comprises of Any Other Ethnic Group, Arab, Chinese

^4^Comprises of Any Other Mixed Background, White and Asian, White and Black African, White and Black Caribbean, Any Other Mixed/Multiple Ethnic Background

^5^Comprises of African, Any Other Black Background, Caribbean

### Uptake of COVID-19 vaccination in pregnancy

**Fig. 2:**
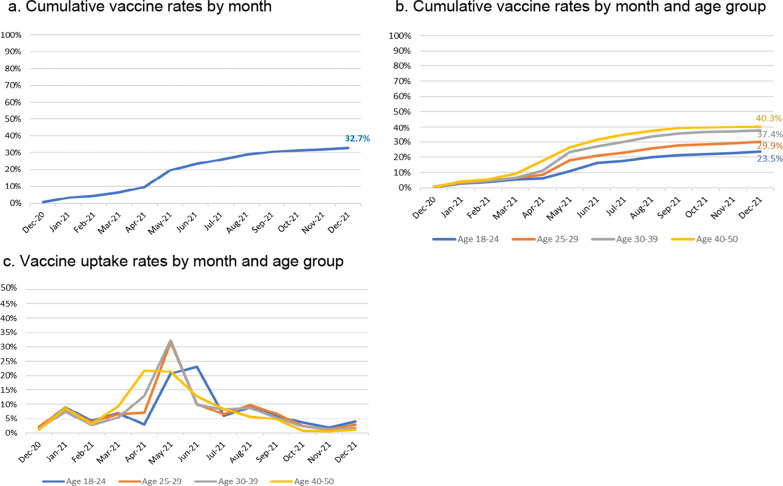
**a** Cumulative vaccine rates by month. **b** Cumulative vaccine rates by month and age group. **c** Vaccine uptake rates by month and age group

Over the study period, 8203 (32.7%) of pregnant women received at least one dose of the COVID-19 vaccine during pregnancy, 8572 (34.1%) were not vaccinated, and 8336 (33.2%) received the vaccine after giving birth. Figure 2a shows that from the start of the vaccination programme on 7th December 2020, there was a slow growth in the uptake of the vaccine among pregnant women. Uptake of the vaccine rose rapidly in April 2021; thus, 32.7% of pregnant women were vaccinated by the end of December 2021. The vaccine uptake each month was consistently lower in younger women < 30 years compared to those aged 30 or older. Overall, only 23.5% of those aged 18–24 were vaccinated by the end of December 2021 compared to 40.3% in those aged 40–50 (Fig. 2b, Additional file 1: Table S3). Starting from April, vaccine uptake rates started rising rapidly among those aged 40–50 with 21.7% of them receiving the vaccine, followed by those aged 25–29 and 30–39 rising rapidly in May (31.7% and 32.4% respectively), and then in June for those aged 18–24 (23.1%). Uptake rates were slower thereafter for all groups (Fig. 2c, Additional file 1: Table S3).

**Fig. 3:**
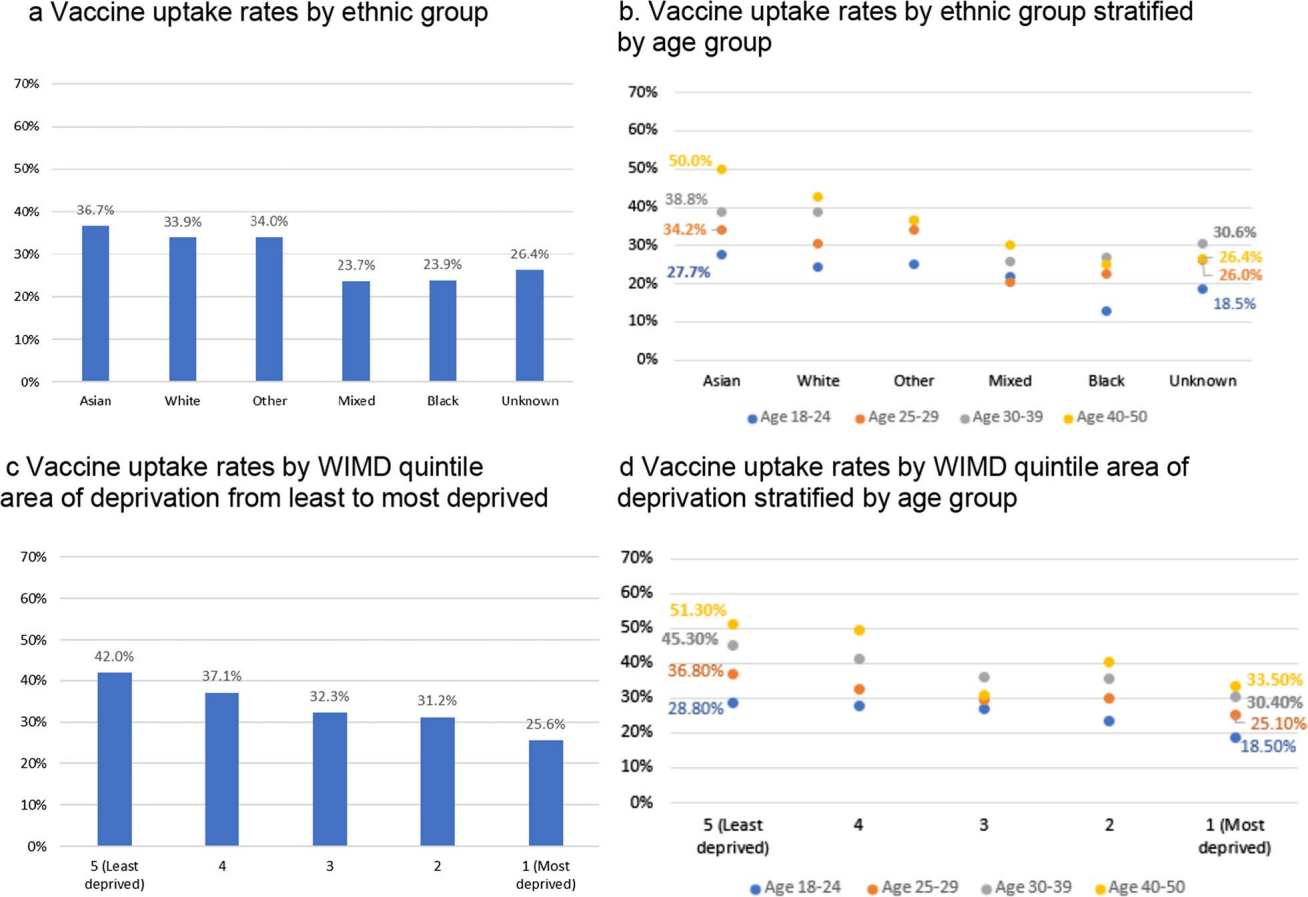
**a** Vaccine uptake rates by ethnic group. **b** Vaccine uptake rates by ethnic group stratified by age group. **c** Vaccine uptake rates by WIMD quintile area of deprivation from least to most deprived. **d** Vaccine uptake rates by WIMD quintile area of deprivation stratified by age group

The uptake rate was higher among Asian women (36.7%, 95% CI 33.6 to 39.8) compared to White women (33.9%, 95% CI 33.2 to 34.5) and the Other ethnic group (34.0%, 95% CI 30.1 to 37.9), especially compared to women of Mixed (23.7%, 95% CI 19.0 to 28.4) or Black ethnicity (23.9%, 95% CI 19.9 to 27.8), where less than a quarter of women had the vaccine (Fig. 3a). The uptake for those aged 18–24 in the Black ethnic group was 14.7% lower than those aged 18–24 in the Asian ethnic group, and 25% lower compared to their peers aged 40–50. Figure 3b shows that the uptake was highest among Asian women and lowest among Black and Mixed groups for all age groups. The uptake rate for those living in the most deprived area is 16.4% lower than those living in the least deprived area. The biggest difference is in those aged 30 or older. In the 30–39 and 40–50 age groups there are 14.9% and 17.8% difference between the most and least deprived areas even though uptake in general is higher in those groups (Fig. 3c, d, Additional file 1: Table S4).

### Examining time to first vaccination in pregnancy

**Fig. 4:**
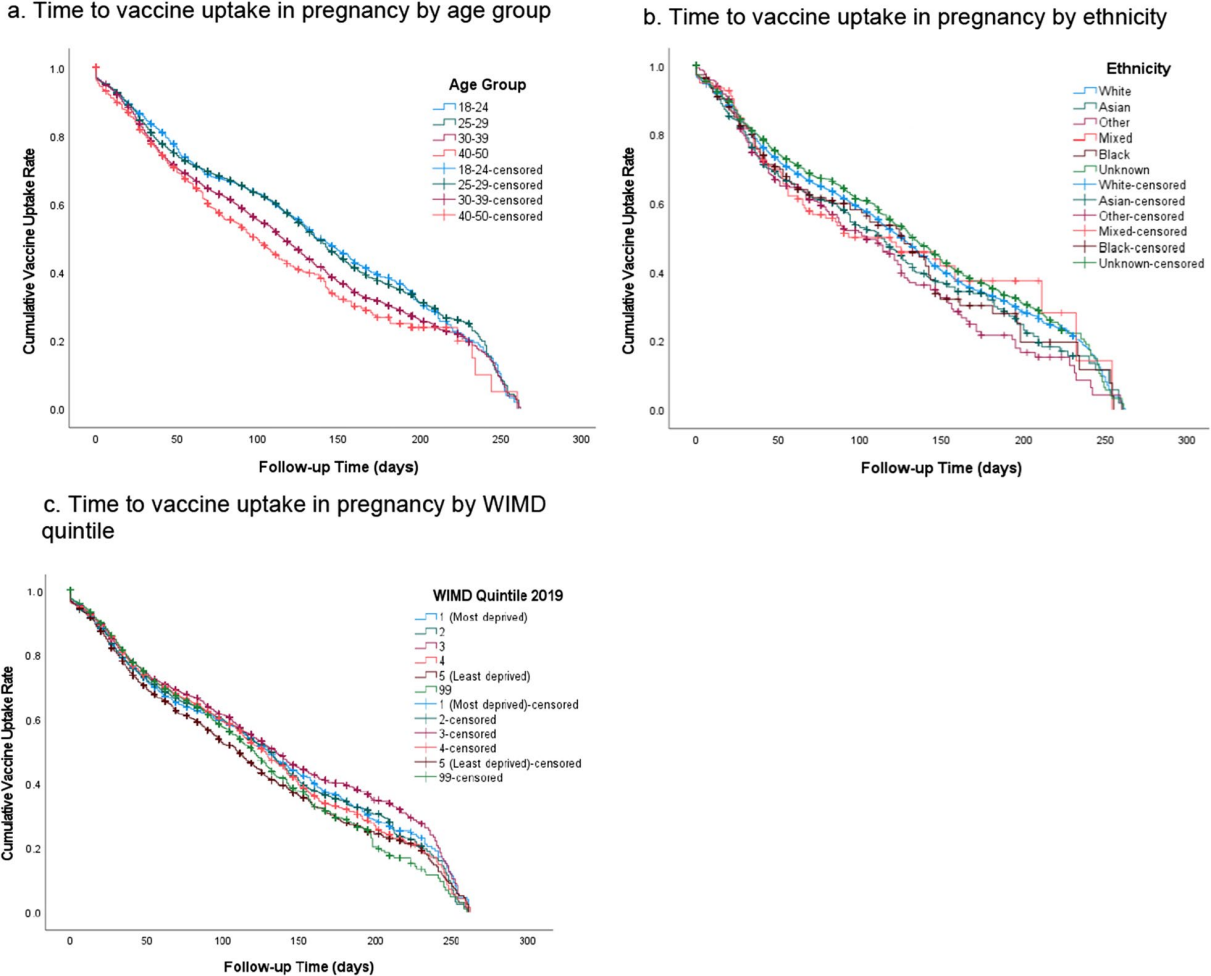
**a** Time to vaccine uptake in pregnancy by age group. **b** Time to vaccine uptake in pregnancy by ethnicity. **c** Time to vaccine uptake in pregnancy by WIMD quintile

Kaplan–Meier survival analysis shows that women aged 18–14 and 25–29 had identical median times to vaccine uptake of 136 days (95% CI 128.5 to 143.5), and 136 days (95% CI 131.6 to 140.4) respectively. This was longer than those aged 30–39 or 40–50, which had median times to vaccine uptake of 115 days (95% CI 111.6 to 118.4) and 99 days (95% CI 87 to 111) respectively. A log rank test was conducted to determine if there were differences in the survival distributions of vaccine uptake times for the different groups. The survival distributions were statistically significantly different, X^2^(3) = 72.5, p < 0.001. Pairwise log rank comparisons were conducted to determine which groups had different survival distributions. There were statistically significant differences between women aged 18–24 compared to those aged 30–39, X^2^(1) = 30.3, p < 0.001, and 18–24 compared to women aged 40–50, X^2^(1) = 26.7, p < 0.001. The same is mirrored in age group 25–29 compared to age groups 30–39 and 40–50. However, the survival distributions for groups 18–24 vs. 25–29 and 30–39 vs. 40–50 were not significantly different (Fig. 4a, Additional file 1: Table S5). The survival distributions between certain ethnic groups were significantly different, X^2^(5) = 16.7, p = 0.005. The Asian and Other ethnic groups had median times of 113 days (95% CI 100.3 to 125.7), and 103 days (95% CI 85.4 to 120.6), which were less than the White’s median time of 125 days (95% CI 122.4 to 127.6). These differences were significant between the Asian vs. the White groups, X^2^(1) = 4.2, p = 0.04, and the Other vs. the White group, X^2^(1) = 6.4, p = 0.01 (Fig. 4b, Additional file 1: Table S5). Those who are living in the most deprived area had a median time to vaccine uptake of 129 days (95% CI 123 to 135.1). This was longer than those living in the least deprived area, which had the lowest median time of 109 days (95% CI 103.2 to, 114.8). The survival distributions between the different deprivation levels were significantly different, X^2^(5) = 41.9, p < 0.001. There were significant differences between those living in the least deprived areas and those living in the most deprived area X^2^(1) = 17.5, p < 0.001, and all the other areas of deprivation (p < 0.001 for all) (Fig. 4c, Additional file 1: Table S5).

### Examining the impact of age, ethnic group, and deprivation area on vaccine uptake

Multivariate Cox regression was conducted to examine the variations in uptake by age group, ethnic group, and area of deprivation jointly on vaccination acceptance. Those aged 40–50 were 1.33 times more likely to have the vaccine compared to those aged 25–29 (HR = 1.33, 95% CI 1.18 to 1.49, p < 0.001), also those aged 30–39 were 1.17 times more likely to have the vaccine compared to those aged 25–29 (HR = 1.17, 95% CI 1.11 to 1.23, p < 0.001) (Table 2). The Asian and Other (the majority of other were Chinese) ethnic groups were 1.12 and 1.18 times more likely to have the vaccine compared to the White group (HR = 1.12, 95% CI 1.00 to 1.25, p = 0.047) and (HR = 1.18, 95% CI 1.03 to 1.37, p = 0.021) respectively. It was also observed that the vaccine uptake was lower among those living in the most deprived areas compared to those living in the most affluent areas (HR = 0.88, 95% CI 0.82 to 0.95, p < 0.001).

**Table 2:** Cox Regression analysis of factors associated with vaccination uptake among pregnant women eligible for vaccination, adjusted analysis

Characteristic		HR^1^ (95% CI^2^)	*P* value^3^
Age group	25–29	Reference	
	18–24	0.99 (0.92–1.07)	0.796
	30–39	1.17 (1.11–1.23)	< 0.001
	40–50	1.33 (1.18–1.49)	< 0.001
Ethnic group	White	Reference	
	Asian	1.12 (1.00–1.25)	0.047
	Other	1.18 (1.03–1.37)	0.021
	Mixed	1.02 (0.81–1.29)	0.855
	Black	1.08 (0.89–1.31)	0.440
	Unknown	0.93 (0.87–1.003)	0.060
WIMD quintile 2019	5th (Least deprived)	Reference	
	4th	0.90 (0.83–0.96)	0.003
	3rd	0.81 (0.76–0.88)	< 0.001
	2nd	0.91 (0.84–0.98)	0.008
	1st (Most deprived)	0.88 (0.82–0.95)	< 0.001

^1^Hazard Ratio, ^2^Confidence Interval (95%), ^3^Significance level accepted at < 0.05

### Mothers views of COVID-19 vaccination in pregnancy

There were 331 women who had a baby during the pandemic or who were currently pregnant between the 1st November 2021 and 24th March 2022 and participating in Born-In-Wales. 44.4% of the women were aged between 30 and 39 and the majority were White (82.2%) (Additional file 1: Table S6). 224 (68%) of women said they would be happy to have the vaccine in pregnancy and 107 (32%) said they would not have the vaccine in pregnancy. Two key themes were developed from the qualitative data: (1) Happy to have the vaccine with sub-themes protecting self and baby from COVID-19, analysis of risk level and sufficient evidence and advice. (2) Concerns about the vaccine with sub-themes lack of research about outcomes of the vaccine for the baby, anxious about COVID-19 and the vaccine, change in advice and information, would not have the vaccine, prefer to wait until later in pregnancy or after pregnancy (Table 3, Additional file 1: Table S7).

**Table 3:** Themes emerging from responses to the question ‘What is your view on having the COVID-19 vaccination in pregnancy, have you or would you have the COVID-19 vaccination when pregnant and why?’

Happy to have the vaccination:
‘When I was pregnant, the advice was not to have it, so I didn't. However, the advice changed soon after I gave birth. If I was pregnant now, with the advice as it is, I would definitely have it so as to give myself protection against the virus in order to keep my baby safe.’ *Respondent 32* ‘Definitely would have had it to protect myself and pass the antibodies onto my unborn child. I know of too many pregnant women who have had covid.’ *Respondent 21* ‘I thought it was important to receive it to protect my baby, family, patients and staff in work.’ *Respondent 3* ‘I am fully vaccinated, I felt it was the best option to protect both myself, my baby and everyone around me’ *Respondent 136* ‘I believe it’s very important to have the vaccine even in pregnancy, I had the first dose at 8 weeks and the second at 16. The added risk of complications and hospital admission in the third trimester are not worth the risk. Plus there is the other potential benefit of some immunity passing on to baby’. *Respondent 169* ‘Had it during pregnancy. Wanted to ensure I was protected and to hopefully pass antibodies onto baby’. *Respondent 51* ‘I would have it as the risk of complications related to the vaccine are a lot lower than the risks to me and unborn baby if caught covid and had a severe case.’ *Respondent 36* ‘I had both doses whilst pregnant. I figured the risks of catching it were worse than the risks of having the vaccine.’ *Respondent 125*
Concerns around the vaccine
‘It’s very hard to make the decision. Obviously, I would not want to catch covid and having the vaccine would reduce that chance. However, because of the initial information to not have the vaccine when pregnant it would make me more cautious. I think I would like to read up on the vaccines before and weigh up the risk vs benefit.’ *Respondent 43* ‘I delayed my vaccination until after the first trimester. I have now had both vaccinations and feel a lot safer.’ *Respondent 124* ‘I have had my first vaccine (I was 28 weeks pregnant) and having my second vaccine next week. This is a very personal choice and having it or not having it should be down to the pregnant woman and her individual research and beliefs.’ *Respondent 110*
Would not choose to have the vaccine
‘I wouldn’t have it as there no long term data available as to how this may or may not affect a baby either in utero or later in life.’ *Respondent 39* ‘Declined vaccine—not enough evidence it is safe for baby.’ *Respondent 103* ‘I personally wouldn't have cos there's no evidence on what it does to unborn but I had it only 2 to 5 months before getting pregnant so suppose there's still risks we may not know about.’ *Respondent 149* ‘I have not had the COVID-19 vaccine during my pregnancy. Although there is evidence to support its use during pregnancy I personally feel that it is too early to see any affects it could have on my child in their future.’ *Respondent 111* ‘I have not had my covid vaccination during pregnancy as I don’t believe there is enough evidence regarding that both myself and my baby will be fine.’ *Respondent 194* ‘No. I would have been too worried as to what the affects may have been on my baby. Not enough research over time that I have seen gave me the confidence that I would risk it. Self-isolation, I feel was safer than having a vaccine that I was not 100% on. If in the future after more people have received the vaccine it was offered and I could see many pregnant women had given birth and the babies had developed with no issues then I would reconsider.’ *Respondent 7* ‘I probably wouldn’t because it’s so new and I would isolate and have it later, which is pretty much what did happen with me. Purely to err on the safe side.’ *Respondent 40*

The main reasons provided for COVID-19 acceptance were to protect the mother and baby, the positives of the vaccine outweigh any potential negatives of having it, and that there has been a satisfactory amount of research conducted on its safety for pregnant women. On the other hand, the main reasons for refusing the vaccine included anxiety over the decision and any possible long-term effects, more research is needed to confirm safety for pregnant women, and preference to wait until after the pregnancy or much later than the first trimester.

## Discussion

This study describes the uptake rates of the COVID-19 vaccination and reasons for vaccine hesitancy or vaccine acceptance in pregnant women in Wales. From the linked data, 34.1% of pregnant women chose not to have the vaccine, 32.7% of the cohort received the vaccine in pregnancy and 33.2% had the vaccine after their baby was born. These findings reflect what was observed in qualitative responses where 31% of pregnant women responding stated that they would not have the COVID-19 vaccine during pregnancy. These findings are similar to the overall high vaccine rates in the UK population where as of March 2022, 78.5% of the UK population has had at least one dose of the COVID-19 vaccine [18]. Across the world there is variation in vaccine uptake with 80.7% of the population has had at least one dose of the vaccine in France, 85.7% in Italy, 77.4% in Germany, and 77.1% in the US [18].

This study found that the decision whether to accept the COVID-19 vaccine during pregnancy was not straightforward and various factors influenced this decision. Expectant mothers described worry and anxiety regarding the vaccine as well as long term concerns for their child. These results are in line with other recent studies reporting anxiety, stress, and vaccine hesitancy in pregnant women during the COVID-19 pandemic [3, 4]. From the survey responses, many expectant mothers described the importance of protecting themselves and their unborn baby and the benefits of receiving the vaccination and deciding to be vaccinated as the benefits outweighed the costs. Understanding the sources of uncertainty regarding the acceptance of vaccines during pregnancy may help with future vaccination strategies to reach pregnant women.

The qualitative results highlighted reasons for hesitancy including concerns over long term safety to the baby and confusion regarding changing recommendations. However, those who were happy to have the vaccine felt it offered protection for their unborn baby and themselves, they felt it might help pass antibodies onto their unborn child and felt the chance of complications and hospital admission were not worth the risk. However, some women were more cautious as guidelines had changed and said they would want to read up more to understand the risk and benefits. Others felt that it was a very personal choice and should be up to the pregnant women and that it was difficult as there was a lot of misinformation and changes in advice was confusing. Those who would not be happy to have the vaccine predominately felt there was not enough long-term data available especially regarding babies’ safety. They felt self-isolation was better protection and a number of women felt it was better to wait until after the birth. These findings are in step with previous vaccine hesitancy research studies [4, 19].

There are changing attitudes over time. For example, a literature review conducted in 2020 indicated that there were high levels of uncertainty regarding the vaccine [19], which may highlight higher levels of vaccine hesitancy compared to now as more research has been conducted regarding the safety of the vaccine. It also included reasons why some women were not hesitant and were pro-vaccination which could potentially inform how to address the hesitancy of others.

From the linked data, age, ethnic group, and deprivation level appeared to influence whether expectant mothers chose to have the vaccine or not and this reflects patterns of uptake in the general population. The youngest age group (age 18–24) were least likely to have the vaccine and the oldest group (age 40 +) were most likely to have the vaccine. Research has found that being younger is associated with both refusal and delay of the COVID-19 vaccine in Portugal [20]. Moreover, studies have indicated evidence of reduced vaccine uptake in younger women aged < 30 who gave birth in London between 1st March, 2020, and 4th July, 2021 [21]. Vaccine hesitancy was also higher in younger age groups (26.5% in 16–24 year olds compared to 4.5% in those aged 75 +) [22]. Vaccine uptake was substantially lower in pregnant women in Scotland than in the general female population; 32.3% of pregnant women compared to 77.4% in all women [8].

The rate of vaccine uptake differed significantly between certain ethnic groups. Asian and Other (e.g., Chinese ethnicities predominantly) were most likely to have the vaccine and differed significantly from those of White ethnic group. Research has found that one of the highest acceptance rates was observed in China, with an average of 77.4% of women accepting a future vaccine during pregnancy [22] which may explain our findings of higher vaccine acceptance in Asian women. In the Black and Mixed ethnic groups, vaccination rates were the lowest. Willingness to be vaccinated was generally high across the UK population [23]. However, vaccine hesitancy does exist in population subgroups. Black and Pakistani/Bangladeshi ethnic groups had higher levels of vaccine hesitancy from responses to a survey [23].

This research showed those living in the least deprived areas in Wales were more likely to have the COVID-19 vaccine compared to those living in the most deprived areas. The characteristics of recipients of the COVID-19 vaccine in England have also been investigated [24]. Research found that there were differences in vaccination uptake in various subgroups including ethnic groups (White 42.5% vaccinated, Black 20.5% vaccinated) and deprivation level (least deprived 44.7% vaccinated, most deprived 37.9% vaccinated) [24]. Similarly, there was evidence of reduced vaccine uptake in younger pregnant women with high levels of deprivation in the UK [21].

## Strengths and limitations

The study has several strengths, it utilises primary and secondary health care data for pregnant women in Wales including the maternity and child health data, it gives a national perspective of COVID-19 vaccine hesitancy, making the findings generalisable due to its total population cohort. The qualitative survey questions allowed a free text response asking participants to provide their opinion on the vaccine and any reasons why they would or would not have it. These responses gave a true insight into the thoughts and feelings of pregnant women in Wales during the pandemic. Findings showing that the reasons for not wanting a vaccine included anxiety about the vaccine, change in advice and information or prefer to delay until after the birth. The mixed methods design used in this study provided rich, detailed information firstly about population-level vaccination uptake rates as well as rich qualitative responses from a cross-section of pregnant women in Wales. Using the two methods provided insight into the reasons why 34.1% of pregnant women refused the vaccine completely and may inform vaccine strategies moving forward.

The study had some limitations, such as not indicating in which trimester pregnant women had the vaccine as it has been reported that pregnant women in the first trimester expressed higher acceptance of COVID-19 vaccination than those in the second and third trimesters [11]. From the qualitative responses, expectant mothers expressed that they wanted to wait until later in their pregnancies before accepting the vaccine. Some commented that they would even wait until after childbirth. The preference of accepting the vaccine after birth was reflected in the quantitative analysis, where 33.2% of pregnant women had the vaccine after childbirth. The survey was based on a convenience sample which lacks a clear generalizability. Another limitation is that recruitment of pregnant women via social media may be prone to selection bias compared to traditional methods of recruitment.

## Conclusion

In conclusion, it is critical to develop tailored strategies to increase the acceptance rates of the COVID-19 vaccine and decrease hesitancy. A more targeted approach to vaccinations may need to be addressed to reach certain groups such as younger people, Black and Mixed ethnic groups, and those living in more deprived areas. Encouraging vulnerable populations, including pregnant women is a priority moving forward.

## Supplementary Information

**Additional file 1.** COVID-19 vaccination in pregnancy.

## Data Availability

The data that support the findings of this study are available from SAIL, but restrictions apply to the availability of these data, which were used under license for the current study, and so are not publicly available. Data are however available from the authors upon reasonable request and with permission of SAIL.

## Abbreviations

EHR: Electronic health records
SAIL: Secure Anonymised Information Linkage
HR: Hazard Ratios
SAGE: The Scientific Advisory Group for Emergencies
JCVI: The UK’s Joint Committee on Vaccination and Immunisation
GP: General Practitioners
WLGP: Wales Longitudinal General Practice
PEDW: Patient Episode Database for Wales
OPDW: Outpatient Database for Wales
NCCH: National Community Child Health
MIDS: Maternal Indicators Dataset
CVVD: COVID-19 Vaccination Data
WDSD: Welsh Demographic Service Dataset
WIMD: Welsh Index for Multiple Deprivation
MD: Median Times
SQL: Structured Query Language
SPSS: Statistical Package for Social Sciences
WHO: The World Health Organisation
IGRP: Information Governance Review Panel
HDR: Health Data Research
BHF: British Heart Foundation
UK: United Kingdom
