# Supplementary material for: COVID-19 vaccination in pregnancy: views and vaccination uptake rates in pregnancy, a mixed methods analysis from SAIL and the Born-In-Wales Birth Cohort
Source: BMC Infect Dis. 2022 Dec 12;22:932. doi: 10.1186/s12879-022-07856-8 (PMC9742024; doi:10.1186/s12879-022-07856-8)
Supplement: Supplementary file 1 — Additional file 1. COVID-19 vaccination in pregnancy. [file 12879_2022_7856_MOESM1_ESM.docx]

Supplementary table 1: Read codes (v2) used to identify pregnancy from the primary care data (GP)

| **Read code** | **Description** |
| --- | --- |
| 13H7. | Unwanted pregnancy |
| 2711. | O/E - fundus 12-16 week size |
| 2712. | O/E - fundus 16-20 week size |
| 2713. | O/E - fundus 20-24 week size |
| 2714. | O/E - fundus 24-28 week size |
| 2715. | O/E - fundus 28-32 week size |
| 2717. | O/E - fundus 34-36 week size |
| 4453. | Serum pregnancy test positive |
| 4654. | Urine pregnancy test positive |
| 584.. | Ultrasound in obstetric diagnosis |
| 5841. | U-S obstetric scan requested |
| 5842. | U-S obstetric scan normal |
| 584B. | Viability US scan |
| 584C. | Antenatal ultrasound result received |
| 584D. | Antenatal ultrasound confirms intra-uterine pregnancy |
| 584Z. | U-S obstetric diagnosis scan NOS |
| 62... | Patient pregnant |
| 621.. | Patient currently pregnant |
| 6212. | Pregnant - blood test confirms |
| 6214. | Pregnant - on history |
| 6216. | Pregnant - planned |
| 6217. | Pregnant - unplanned - wanted |
| 621C. | Unplanned pregnancy |
| 621Z. | Patient pregnant NOS |
| 622.. | Antenatal care: gravida No. |
| 623.. | A/N care: obstetric risk |
| 625Z. | A/N care: social risk NOS |
| 628Z. | A/N risk NOS |
| 62A.. | A/N care provider |
| 62A3. | A/N - shared care |
| 62B.. | Delivery booking place |
| 62B3. | G.P. unit delivery booking |
| 62B4. | Consultant unit booking |
| 62B8. | Midwife unit delivery booking |
| 62C.. | Delivery booking - length of stay |
| 62F.. | Antenatal amniocentesis |
| 62G.. | Antenatal ultrasound scan |
| 62GB. | Antenatal ultrasounds scan at 4-8 weeks |
| 62GZ. | Antenatal ultrasound scan NOS |
| 62L.. | Antenatal blood group screen |
| 62N.. | Antenatal examinations |
| 62N1. | A/N booking examination |
| 62N3. | A/N 16-week examination |
| 62O1. | Fetal movements felt |
| 62X.. | Length of gestation |
| 62Y.. | Routine antenatal care |
| 62a.. | Pregnancy review |
| 62b.. | Antenatal HIV screening |
| 62c.. | Antenatal screening |
| 6776. | Pregnancy termination counselling |
| 679E. | Antenatal education |
| 7F051 | Diagnostic amniocentesis |
| 7F060 | Cerclage of cervix of gravid uterus |
| 7F25. | Obstetric monitoring |
| 7F2B1 | Ultrasound monitoring of early pregnancy |
| 8B75. | Vitamin supplement - pregnancy |
| 8H7W. | Refer to TOP counselling |
| 8HHV. | Referral for termination of pregnancy |
| 8HHf. | Refer to early pregnancy unit |
| 8HT9. | Referral to antenatal clinic |
| 8M6.. | Requests pregnancy termination |
| 95... | Maternity services admin. |
| 9N1N. | Seen in antenatal clinic |
| L1... | Pregnancy complications |
| L10.. | Haemorrhage in early pregnancy |
| L100. | Threatened abortion |
| L10y. | Other haemorrhage in early pregnancy |
| L11.. | Antepartum haemorrhage, abruptio placentae, placenta praevia |
| L1246 | Pre-eclampsia, unspecified |
| L13.. | Excessive pregnancy vomiting |
| L130. | Mild hyperemesis gravidarum |
| L1300 | Mild hyperemesis unspecified |
| L13y. | Other pregnancy vomiting |
| L13z. | Unspecified pregnancy vomiting |
| L1668 | Urinary tract infection complicating pregnancy |
| L16y5 | Abdominal pain in pregnancy |
| L1808 | Diabetes mellitus arising in pregnancy |
| L1809 | Gestational diabetes mellitus |
| L1825 | Iron deficiency anaemia of pregnancy |
| L18A0 | Cholestasis of pregnancy |
| L210. | Twin pregnancy |
| L25.. | Known or suspected fetal abnormality |
| L264. | Intrauterine death |
| L280. | Oligohydramnios |
| L33z. | Umbilical cord complications NOS |
| L413. | Antenatal deep vein thrombosis |
| L510. | Maternal care for hydrops fetalis |
| Lyu21 | [X]Other vomiting complicating pregnancy |
| Lyu25 | [X]Other specified pregnancy-related conditions |
| Z212. | Antenatal care |
| Z22.. | Pregnancy observations |
| Z225. | Normal pregnancy |
| Z226. | Pregnancy problem |
| Z227. | Confirmation of pregnancy |
| Z2291 | Intrauterine pregnancy |
| Z22A. | Observation of pattern of pregnancy |
| Z22A1 | Low risk pregnancy |
| Z22A4 | Early stage of pregnancy |
| Z22AA | Wanted pregnancy |
| Z22AB | Unplanned pregnancy |
| Z22B1 | Single pregnancy |
| Z22C1 | Estimated date of delivery from last period |
| Z22C3 | Length of gestation |
| Z22D1 | Viable pregnancy |
| Z22D2 | Non-viable pregnancy |
| Z22D3 | Uncertain viability of pregnancy |
| ZV222 | [V]Pregnancy confirmed |
| ZV223 | [V]Pregnant state, incidental |
| ZV231 | [V]Pregnancy with history of trophoblastic disease |
| ZV28. | [V]Antenatal screening |
| 1531. | Last menstural period-1st day |
| 271B. | O/E - fundal size = dates |
| 2726. | O/E - fetal presentation unsure |
| 2766. | O/E - fetal heart 120-160 |
| 5391. | Obstetric X-ray - fetus |
| 584A. | Dating/Booking US scan |
| 67A.. | Pregnancy advice |
| 67A2. | Diet in pregnancy advice |
| 67A3. | Pregnancy smoking advice |
| 67AE. | Folic acid advice in first trimester of pregnancy |
| 67AF. | Pregnancy advice for patients with epilepsy |
| 7F261 | Viability scan |
| 7F2B. | Obstetric ultrasound monitoring |
| 9511. | FP24 signed by patient |
| 957.. | FW 8-applic for presc exempt |
| 9kv.. | Pertussis vaccination programme pregnant women enhance service admin |
| 9mK.. | Pertussis vaccination in pregnancy invitation |
| 9Nk3. | Seen in fetal medicine clinic |
| 9NkN. | Seen in early pregnancy unit |
| 9NV1. | Antenatal clinic |
| ZV286 | [V] Antenatal screening for chromosomal anomalies |
| 6556. | Pretussis vaccination in pregnancy |

Supplementary table 2: ICD-10 version:2019 codes used to identify pregnancy from the hospital admissions data (PEDW)

| **ICD-10 code** | **Definition** |
| --- | --- |
| O11X | Pre-eclampsia superimposed on chronic hypertension |
| O120 | Gestational oedema |
| O121 | Gestational proteinuria |
| O122 | Gestational oedema with proteinuria |
| O13X | Gestational [pregnancy-induced] hypertension |
| O140 | Mild to moderate pre-eclampsia |
| O141 | Severe pre-eclampsia |
| O142 | HELLP syndrome |
| O149 | Pre-eclampsia, unspecified |
| O150 | Eclampsia in pregnancy |
| O116X | Unspecified maternal hypertension |
| O200 | Threatened abortion |
| O208 | Other haemorrhage in early pregnancy |
| O209 | Haemorrhage in early pregnancy, unspecified |
| O20 | Haemorrhage in early pregnancy |
| O210 | Mild hyperemesis gravidarum |
| O211 | Hyperemesis gravidarum with metabolic disturbance |
| O212 | Late vomiting of pregnancy |
| O219 | Vomiting of pregnancy, unspecified |
| O220 | Varicose veins of lower extremity in pregnancy |
| O223 | Deep phlebothrombosis in pregnancy |
| O224 | Haemorrhoids in pregnancy |
| O228 | Other venous complications in pregnancy |
| O229 | Venous complication in pregnancy, unspecified |
| O230 | Infections of kidney in pregnancy |
| O234 | Unspecified infection of urinary tract in pregnancy |
| O235 | Infections of the genital tract in pregnancy |
| O239 | Other and unspecified genitourinary tract infection in pregnancy |
| O244 | Diabetes mellitus arising in pregnancy |
| O249 | Diabetes mellitus in pregnancy, unspecified |
| O260 | Excessive weight gain in pregnancy |
| O261 | Low weight gain in pregnancy |
| O262 | Pregnancy care of habitual aborter |
| O265 | Maternal hypotension syndrome |
| O268 | Other specified pregnancy-related conditions |
| O269 | Pregnancy-related condition, unspecified |
| O280 | Abnormal haematological finding on antenatal screening of mother |
| O281 | Abnormal biochemical finding on antenatal screening of mother |
| O283 | Abnormal ultrasonic finding on antenatal screening of mother |
| O289 | Abnormal finding on antenatal screening of mother, unspecified |
| O300 | Twin pregnancy |
| O301 | Triplet pregnancy |
| O320 | Maternal care for unstable lie |
| O321 | Maternal care for breech presentation |
| O322 | Maternal care for transverse and oblique lie |
| O324 | Maternal care for high head at term |
| O326 | Maternal care for compound presentation |
| O328 | Maternal care for other malpresentation of fetus |
| O329 | Maternal care for malpresentation of fetus, unspecified |
| O340 | Maternal care for congenital malformation of uterus |
| O341 | Maternal care for tumour of corpus uteri |
| O342 | Maternal care due to uterine scar from previous surgery |
| O343 | Maternal care for cervical incompetence |
| O344 | Maternal care for other abnormalities of cervix |
| O346 | Maternal care for abnormality of vagina |
| O347 | Maternal care for abnormality of vulva and perineum |
| O348 | Maternal care for other abnormalities of pelvic organs |
| O350 | Maternal care for (suspected) central nervous system malformation in fetus |
| O351 | Maternal care for (suspected) chromosomal abnormality in fetus |
| O352 | Maternal care for (suspected) hereditary disease in fetus |
| O358 | Maternal care for other (suspected) fetal abnormality and damage |
| O359 | Maternal care for (suspected) fetal abnormality and damage, unspecified |
| O35 | Maternal care for known or suspected fetal abnormality and damage |
| O360 | Maternal care for rhesus isoimmunization |
| O361 | Maternal care for other isoimmunization |
| O363 | Maternal care for signs of fetal hypoxia |
| O364 | Maternal care for intrauterine death |
| O365 | Maternal care for poor fetal growth |
| O366 | Maternal care for excessive fetal growth |
| O368 | Maternal care for other specified fetal problems |
| O369 | Maternal care for fetal problem, unspecified |
| O40X | Polyhydramnios |
| O410 | Oligohydramnios |
| O418 | Other specified disorders of amniotic fluid and membranes |
| O429 | Premature rupture of membranes, unspecified |
| O438 | Other placental disorders |
| O440 | Placenta praevia specified as without haemorrhage |
| O441 | Placenta praevia with haemorrhage |
| O459 | Premature separation of placenta, unspecified |
| O468 | Other antepartum haemorrhage |
| O469 | Antepartum haemorrhage, unspecified |
| O470 | False labour before 37 completed weeks of gestation |
| O471 | False labour at or after 37 completed weeks of gestation |
| O479 | False labour, unspecified |
| O48X | Prolonged pregnancy |
| O718 | Other specified obstetric trauma |
| O882 | Obstetric blood-clot embolism |
| Z321 | Pregnancy confirmed |
| Z33X | Pregnant state, incidental |
| Z340 | Supervision of normal first pregnancy |
| Z348 | Supervision of other normal pregnancy |
| Z349 | Supervision of normal pregnancy, unspecified |
| Z352 | Supervision of pregnancy with other poor reproductive or obstetric history |
| Z353 | Supervision of pregnancy with history of insufficient antenatal care |
| Z357 | Supervision of high-risk pregnancy due to social problems |
| Z358 | Supervision of other high-risk pregnancies |
| Z368 | Other antenatal screening |
| Z369 | Antenatal screening, unspecified |

Supplementary Table 3. Rates and cumulative rates of vaccine uptake during pregnancy for all vaccinated women by month and age group

| **Age** | **18-24** | | | **25-29** | | | **30-39** | | | **40-50** | | | **All age groups** | | |
| --- | --- | --- | --- | --- | --- | --- | --- | --- | --- | --- | --- | --- | --- | --- | --- |
|  | **n** | **%** | **Cum %** | **n** | **%** | **Cum %** | **n** | **%** | **Cum %** | **n** | **%** | **Cum %** | **N** | **%** | **Cum %** |
| **Dec-20** | 22 | 2.0% | 0.5% | 51 | 2.3% | 0.7% | 75 | 1.7% | 0.6% | <5 | 1.2% | 0.5% | 152 | 1.9% | 0.6% |
| **Jan-21** | 99 | 9.0% | 2.6% | 188 | 8.4% | 3.2% | 335 | 7.4% | 3.4% | 30 | 8.7% | 4.0% | 652 | 7.9% | 3.2% |
| **Feb-21** | 48 | 4.4% | 3.6% | 86 | 3.9% | 4.4% | 131 | 2.9% | 4.5% | 11 | 3.2% | 5.3% | 276 | 3.4% | 4.3% |
| **Mar-21** | 76 | 6.9% | 5.3% | 147 | 6.6% | 6.3% | 246 | 5.4% | 6.5% | 32 | 9.3% | 9.0% | 501 | 6.1% | 6.3% |
| **Apr-21** | 34 | 3.1% | 6.0% | 158 | 7.1% | 8.5% | 585 | 12.9% | 11.3% | 75 | 21.7% | 17.7% | 852 | 10.4% | 9.7% |
| **May-21** | 226 | 20.7% | 10.8% | 705 | 31.7% | 17.9% | 1469 | 32.4% | 23.4% | 74 | 21.4% | 26.4% | 2474 | 30.2% | 19.5% |
| **Jun-21** | 253 | 23.1% | 16.3% | 225 | 10.1% | 20.9% | 444 | 9.8% | 27.1% | 44 | 12.8% | 31.5% | 966 | 11.8% | 23.4% |
| **Jul-21** | 66 | 6.0% | 17.7% | 144 | 6.5% | 22.9% | 370 | 8.2% | 30.1% | 29 | 8.4% | 34.9% | 609 | 7.4% | 25.8% |
| **Aug-21** | 98 | 9.0% | 19.8% | 217 | 9.8% | 25.8% | 401 | 8.8% | 33.4% | 20 | 5.8% | 37.2% | 736 | 9.0% | 28.7% |
| **Sep-21** | 65 | 5.9% | 21.2% | 149 | 6.7% | 27.8% | 246 | 5.4% | 35.4% | 17 | 4.9% | 39.2% | 477 | 5.8% | 30.6% |
| **Oct-21** | 41 | 3.7% | 22.0% | 55 | 2.5% | 28.5% | 104 | 2.3% | 36.3% | <5 | 0.9% | 39.6% | 203 | 2.5% | 31.5% |
| **Nov-21** | 22 | 2.0% | 22.5% | 32 | 1.4% | 29.0% | 51 | 1.1% | 36.7% | <5 | 0.6% | 39.8% | 107 | 1.3% | 31.9% |
| **Dec-21** | 44 | 4.0% | 23.5% | 68 | 3.1% | 29.9% | 82 | 1.8% | 37.4% | <5 | 1.2% | 40.3% | 198 | 2.4% | 32.7% |
| **Total** | **1094** |  |  | **2225** |  |  | **4539** |  |  | **345** |  |  | **8203** |  |  |

Supplementary Table 4. Vaccine uptake by Ethnicity/WIMD quintile of deprivation of area of residence and age group

| **Age group** | **18-24** | | | **25-29** | | | **30-39** | | | **40-50** | | |
| --- | --- | --- | --- | --- | --- | --- | --- | --- | --- | --- | --- | --- |
|  | **Vaccinated** | **All** | **%** | **Vaccinated** | **All** | **%** | **Vaccinated** | **All** | **%** | **Vaccinated** | **All** | **%** |
| **Ethnicity** | | | | | | | | | | | | |
| **Asian** | 28 | 101 | 27.7% | 92 | 269 | 34.2% | 191 | 492 | 38.8% | 20 | 40 | 50.0% |
| **White** | 887 | 3623 | 24.5% | 1763 | 5782 | 30.5% | 3685 | 9482 | 38.9% | 282 | 660 | 42.7% |
| **Other** | 23 | 92 | 25.0% | 52 | 153 | 34.0% | 108 | 296 | 36.5% | 11 | 30 | 36.7% |
| **Mixed** | 13 | 60 | 21.7% | 17 | 83 | 20.5% | 42 | 163 | 25.8% | <5 | 10 | 30.0% |
| **Black** | 7 | 54 | 13.0% | 29 | 128 | 22.7% | 61 | 226 | 27.0% | 8 | 32 | 25.0% |
| **Unknown** | 136 | 734 | 18.5% | 268 | 1032 | 26.0% | 454 | 1482 | 30.6% | 23 | 87 | 26.4% |
| **WIMD_Quintile_2019** | | | | | | | | | | | | |
| **5 (Least deprived)** | 88 | 306 | 28.8% | 332 | 903 | 36.8% | 1024 | 2261 | 45.3% | 80 | 156 | 51.3% |
| **4th** | 141 | 506 | 27.9% | 361 | 1110 | 32.5% | 834 | 2034 | 41.0% | 76 | 154 | 49.4% |
| **3rd** | 197 | 733 | 26.9% | 367 | 1254 | 29.3% | 735 | 2034 | 36.1% | 42 | 136 | 30.9% |
| **2nd** | 251 | 1071 | 23.4% | 447 | 1500 | 29.8% | 743 | 2093 | 35.5% | 53 | 131 | 40.5% |
| **1 (Most deprived)** | 292 | 1582 | 18.5% | 458 | 1828 | 25.1% | 687 | 2257 | 30.4% | 58 | 173 | 33.5% |
| **NA** | 125 | 466 | 26.8% | 256 | 852 | 30.0% | 518 | 1462 | 35.4% | 38 | 109 | 34.9% |

Supplementary Table 5. Chi-square (*P* value) of COVID-19 vaccine uptake by pregnant women characteristics compared.

| **Log Rank** | **X2** | **P value** | **X2** | **P value** | **X2** | **P value** | **X2** | **P value** | **X2** | **P value** | **X2** | **P value** |
| --- | --- | --- | --- | --- | --- | --- | --- | --- | --- | --- | --- | --- |
| **Age** | **18-24** | | **25-29** | | **30-39** | | **40-50** | |  |  |  |  |
| **18-24** | - | - | 0.46 | 0.50 | 30.32 | <.001 | 26.74 | <.001 |  |  |  |  |
| **25-29** | - | - | - | - | 42.73 | <.001 | 26.32 | <.001 |  |  |  |  |
| **30-39** | - | - | - | - | - | - | 4.75 | 0.029 |  |  |  |  |
| **40-50** | - | - | - | - | - | - | - | - |  |  |  |  |
|  |  | |  | |  | |  | |  | |  | |
| **Ethnicity** | **Asian** | | **White** | | **Other** | | **Mixed** | | **Black** | | **Unknown** | |
| **Asian** | - | - | 4.16 | 0.041 | 0.60 | 0.44 | 0.23 | 0.629 | 0.06 | 0.801 | 8.61 | 0.003 |
| **White** | - | - | - | - | 6.44 | 0.011 | 0.10 | 0.775 | 0.39 | 0.531 | 4.65 | 0.031 |
| **Other** | - | - | - | - | - | - | 0.76 | 0.385 | 0.42 | 0.519 | 11.17 | 0.001 |
| **Mixed** | - | - | - | - | - | - | - | - | 0.04 | 0.852 | 1.00 | 0.319 |
| **Black** | - | - | - | - | - | - | - | - | - | - | 1.97 | 0.161 |
| **Unknown** | - | - | - | - | - | - | - | - | - | - | - | - |
|  |  | |  | |  | |  | |  | |  | |
| **WIMD** | **1st** | | **2nd** | | **3rd** | | **4th** | | **5th** | | **Unknown** | |
| **1^st^** | - | - | 0.69 | 0.407 | 3.32 | 0.068 | 0.80 | 0.371 | 17.45 | <.001 | 3.45 | 0.063 |
| **2^nd^** | - | - | - | - | 6.80 | 0.009 | 0.05 | 0.823 | 12.90 | <.001 | 1.70 | 0.192 |
| **3^nd^** | - | - | - | - | - | - | 7.24 | 0.007 | 36.13 | <.001 | 12.51 | <.001 |
| **4^th^** | - | - | - | - | - | - | - | - | 12.24 | <.001 | 1.43 | 0.232 |
| **5^th^** | - | - | - | - | - | - | - | - | - | - | 3.71 | 0.054 |
| **Unknown** | - | - | - | - | - | - | - | - | - | - | - | - |

Supplementary Table 6. Demographics for women completing the survey

|  |  | **N** | **%** |
| --- | --- | --- | --- |
| **Age** | 18-24 | 21 | 6.3 |
|  | 25-29 | 85 | 25.7 |
|  | 30-39 | 147 | 44.4 |
|  | 40-50 | 14 | 4.2 |
|  | Unknown | 64 | 19.4 |
| **Ethnicity** | White | 272 | 82.2 |
|  | Non-White | 5 | 1.5 |
|  | Unknown | 54 | 16.3 |

Supplementary Table 7. Themes and sub themes emerging from responses to the question ‘What is your view on having the COVID vaccination in pregnancy, have you or would you have the COVID vaccination when pregnant and why?’

| **Happy to have the vaccine** |
| --- |
| **Protecting self and baby from COVID-19** |
| ‘Yes, I would definitely have the vaccine to protect me and my baby.’ ***Respondent 37***  ‘I believe it offers better protection to my unborn baby and myself.’ ***Respondent 38***  ‘When I was pregnant, the advice was not to have it, so I didn't. However, the advice changed soon after I gave birth. If I was pregnant now, with the advice as it is, I would definitely have it so as to give myself protection against the virus in order to keep my baby safe.’ ***Respondent 32***  ‘I would have the covid vaccine whilst pregnant. I had it whilst breastfeeding. Pros definitely outweigh any potential cons. I would be happy to pass on some immunity to my baby.’ ***Respondent 28***  ‘I would have had it during pregnancy if it had been available - I feel there is enough evidence to support its use during pregnancy to protect both mum and unborn baby.’ ***Respondent 26***  ‘Due to the information now, I have, and would stress the safety aspect that vaccine does help. I would not want to be pregnant with covid risking my health and the baby.’ ***Respondent 24***  ‘I had mine while breast feeding. I would have had it if pregnant at the time, feel it’s important to be vaccinated for the benefit of myself, partner & children as well as the general public.’ ***Respondent 8***  ‘Definitely would have had it to protect myself and pass the antibodies onto my unborn child. I know of too many pregnant women who have had covid.’ ***Respondent 21***  ‘I would have one while pregnant to keep myself and the baby safe.’ ***Respondent 5***  ‘I thought it was important to receive it to protect my baby, family, patients and staff in work.’ ***Respondent 3***  ‘I am fully vaccinated, I felt it was the best option to protect both myself, my baby and everyone around me’ ***Respondent 136***  ‘I had my second vaccine whilst 9 weeks pregnant as I got told it was safe to do so. I want to be protected as much as I can.’ ***Respondent 151***  ‘I have had my vaccination and hope it offers some protection to me and my baby’ ***Respondent 158***  ‘I believe it’s very important to have the vaccine even in pregnancy, I had the first dose at 8 weeks and the second at 16. The added risk of complications and hospital admission in the third trimester are not worth the risk. Plus there is the other potential benefit of some immunity passing on to baby’ ***Respondent 169***  ‘Yes I would have it whilst pregnant. The more protection the better’ ***Respondent 200***  ‘Had it during pregnancy. Wanted to ensure I was protected and to hopefully pass antibodies onto baby’ ***Respondent 51***  ‘I have had all vaccinations and booster ether before/ during my pregnancy to protect my baby, myself and those around me’ ***Respondent 67***  ‘Very important to encourage everyone to have vaccine to pass vital antibodies on to newborns’ ***Respondent 74***  ‘I’ve had both jabs and the booster. I’d rather be protected and have a chance that the baby is protected if a long term stay in hospital is needed’ ***Respondent 97***  ‘I’ve had the COVID vaccine in pregnancy and think it was the best choice to protect both myself and my baby’ ***Respondent 283*** |
| **Analysis of Risk Level** |
| ‘I would have it as the risk of complications related to the vaccine are a lot lower than the risks to me and unborn baby if caught covid and had a severe case.’ ***Respondent 36***  ‘I’d have it while pregnant because I feel the possible side effects are not as risky as getting covid while pregnant.’ ***Respondent 35***  ‘I would have the vaccination if I was pregnant, as the risk of catching covid and having bad side effects of that is much greater than the risk of something bad happening because of the vaccine.’ ***Respondent 9***  ‘It’s very hard to make the decision. Obviously, I would not want to catch covid and having the vaccine would reduce that chance. However, because of the initial information to not have the vaccine when pregnant it would make me more cautious. I think I would like to read up on the vaccines before and weigh up the risk vs benefit.’ ***Respondent 43***  ‘I have had the COVID vaccination in pregnancy. I read the information showing no additional adverse effects had been shown in pregnant women who had taken the vaccination and compared this with the research showing greater likelihood of illness or death for myself and baby if I caught Covid in late pregnancy.’ ***Respondent 118***  ‘I had both doses whilst pregnant. I figured the risks of catching it were worse than the risks of having the vaccine’ ***Respondent 125***  ‘I am pregnant; I had my booster in pregnancy at 25 weeks gestation. I was apprehensive about the high prevalence of Covid in the population as I entered my third trimester, and working as a GP I was concerned my exposure risks were higher. I was also concerned about potential health risk of contracting Covid in pregnancy for me and baby so felt on balance the benefit of taking the booster far outweighed the potential risks’ Respondent 46  ‘I have taken the booster at 25 weeks as I was concerned about the population prevalence of Covid as I entered the third trimester. I was concerned about the potential health issues for baby and myself if I was to contract Covid in pregnancy. I felt the benefits far outweighed the potential risks’ ***Respondent 139***  ‘I have had both vaccinations for it after lots of research. Due to when I was going to give birth I felt catching covid at this time was a higher risk I didn't want to take.’ ***Respondent 146***  ‘Yes I would. Rather try to stay safe than chance it.’ ***Respondent 16***  ‘Yes I have had both covid vaccinations and would get a booster if sent a letter about it, I know the risks of covid of pregnant woman and would rather be vaccinated than not’ ***Respondent 171***  ‘Yes because I feel the benefits of keeping myself and my baby safe outweigh the risks’ ***Respondent 175***  ‘Known benefits outweigh any potential risks. Have had both in pregnancy’ ***Respondent 196***  ‘I’ve had the first dose, I decided that having the jab was nothing compared to what might happen if I caught covid’ ***Respondent 57*** |
| **Sufficient evidence and advice** |
| ‘I would have the vaccine while pregnant. I understand that a lot of research has gone into producing the vaccines. If it's safe for the majority of people to have it then I'd assume it was safe for pregnant women too as long as they had a straightforward pregnancy and no serious complications.’ ***Respondent 18***  ‘Yes I would have had if the opportunity had been there when I was pregnant. I’ve read research & seen enough evidence to make me happy it would be safe for me & baby.’ ***Respondent 14***  ‘Yes I would have it. I had a baby in 2020 and would have taken a vaccine in a heartbeat.’ ***Respondent 15***  ‘I would have had the vaccine provided it was recommended to do so, as with all other vaccinations.’ ***Respondent 6***  ‘I had the vaccination whilst pregnant. I had no concerns about it and felt that the advice to have it was clear.’ ***Respondent 44***  ‘I delayed my vaccination until after the first trimester. I have now had both vaccinations and feel a lot safer.’ ***Respondent 124***  ‘Had them both before pregnant spoke to midwife and she was happy for me to have the booster’ ***Respondent 141***  ‘I have had both of my vaccinations before I got pregnant but would get them during pregnancy if advised.’ ***Respondent 147***  ‘I'm sure that clear and consistent advice from a health care professional about the benefits of the vaccination with research based evidence regarding safety would benefit’ ***Respondent 11***  ‘I have had my first vaccine (I was 28 weeks pregnant) and having my second vaccine next week. This is a very personal choice and having it or not having it should be down to the pregnant woman and her individual research and beliefs’ ***Respondent 110***  ‘I have had both vaccines and know it is safe’ ***Respondent 181***  ‘Have been vaccinated, believe it is really important for all to be vaccinated, including pregnant women’ ***Respondent 182***  ‘Had my second vaccine when I was about 6 weeks pregnant and trusted the whole process. The people administrating the vaccine were really informative and asked me to consider if I wanted it before having it which I appreciated.’ ***Respondent 192***  ‘I had my second vaccine and booster whilst pregnant as I was advised by my midwife this is the safest option for me and my baby’ ***Respondent 224*** |
| **Concerns about the vaccine** |
| **Lack of research about outcomes of the vaccine for the baby** |
| ‘I wouldn’t have it as there no long term data available as to how this may or may not affect a baby ether in utero or later in life.’ ***Respondent 39***  ‘Absolutely not. There is insufficient data to support the use of the Covid vaccine during pregnancy and the clinical trials are not yet complete.’ ***Respondent 34***  ‘Did not have it when pregnant, too little research in pregnant population. New trial vaccine that I only just about decided to have recently’ ***Respondent 19***  ‘I had given birth before the vaccine was available. I probably would have had the vaccine, but would have been concerned about the lack of research.’ ***Respondent 17***  ‘Declined vaccine - not enough evidence it is safe for baby’ ***Respondent 103***  ‘Not enough research into the effect it will have on the baby.’ ***Respondent 116***  ‘I am double vaccinated already but personally if I wasn’t I wouldn’t have the vaccination due to lack of research & testing on pregnant women’ ***Respondent 131***  ‘No not enough evidence or trials around vaccine and pregnant women’ ***Respondent 134***  ‘I personally wouldn't have cos there's no evidence on what it does to unborn but I had it only 2 to 5 months before getting pregnant so suppose there's still risks we may not know about’ ***Respondent 149***  ‘I have not had the COVID-19 vaccine during my pregnancy. Although there is evidence to support its use during pregnancy I personally feel that it is too early to see any affects it could have on my child in their future.’ ***Respondent 111***  ‘I’d never risk it as we don’t know long term effects’ ***Respondent 137***  ‘I had my vaccines before I got pregnant, there is not enough research on the vaccine in general let alone in pregnancy for long term risks or effects so no I would not have the vaccine or booster whilst pregnant’ ***Respondent 159***  ‘I have not had my covid vaccination during pregnancy as I don’t believe there is enough evidence regarding that both myself and my baby will be fine’ ***Respondent 194***  ‘No I have not received the COVID vaccination due to the lack of scientific data that the vaccination is safe’ ***Respondent 71***  ‘I don’t feel like there’s enough evidence for me to be confident in having it in pregnancy’ ***Respondent 72***  ‘Didn't have it as just not enough evidence it's safe’ ***Respondent 232*** |
| **Anxious about COVID-19 and the vaccine** |
| ‘At the time, I don't think I would have. I was very nervous anyway and this would have pushed me over the edge.’ ***Respondent 30***  ‘I would have had the vaccination when I was pregnant even though I would have been concerned about unknown effects on baby. I was very worried that I may catch the virus and the risk to myself and baby during that time’ ***Respondent 29***  ‘I would more than likely have had the vaccination when pregnant. I'm glad the vaccine wasn't available while I was pregnant as it would have been a difficult decision, just because I was quite anxious about anything going wrong.’ ***Respondent 12***  ‘No. I would have been too worried as to what the affects may have been on my baby. Not enough research over time that I have seen gave me the confidence that I would risk it. Self-isolation, I feel was safer than having a vaccine that I was not 100% on. If in the future after more people have received the vaccine it was offered and I could see many pregnant women had given birth and the babies had developed with no issues then I would reconsider.’ ***Respondent 7***  ‘Yes I had it but have experienced anxiety around it’ ***Respondent 113***  ‘I had the vaccination after pregnancy but as soon as it was offered to me. I would have had the vaccination whilst pregnant. Though I think I would feel anxious about the decision.’ ***Respondent 4***  ‘Was uncomfortable having vaccine while pregnant as long term effects on potential development of the baby is unknown at this stage’ ***Respondent 66*** |
| **Change in Advice and Information** |
| ‘I gave birth before the vaccines were made so I couldn't have it. If I was pregnant now I would have it but there has been a lot of confusion and change with the advice so I understand why a lot of women are scared to have it.’ ***Respondent 27***  ‘Didn't have it in pregnancy as very early on in vaccination drive (offered it when not recommended for pregnant women). Although guidance did subsequently change, I did not get the vaccine until post baby's birth. With increasing evidence to suggest no concerns with the vaccine and risk to pregnant women I would now be more likely to have it.’ ***Respondent 25***  ‘I would have had it but reluctantly so due to concerns about side effects etc., lack of information and lots of misinformation floating around regarding the vaccines whilst pregnant.’ ***Respondent 10***  ‘I did not have the vaccine while I was pregnant (I am 5weeks pp) because from the start it was just so confusing. The info was different and then it changed and the government & media made out as if it was our fault that we hadn’t had it and starting to scare us in to having it.’ ***Respondent 45***  ‘I did not have the vaccine when pregnant. Purely because when I found out I was pregnant, the advice was not to have the vaccine. Although this advice changed throughout the pregnancy I decided to wait as I found a lack of evidence’ ***Respondent 150***  ‘I received my first vaccine before I got pregnant and was due my second while I was pregnant and had very mixed information which made me not get my second’ ***Respondent 191***  ‘I didn’t have it as the advice had changed 3 times since I was pregnant’ ***Respondent 258*** |
| **Would not have the vaccine** |
| ‘No its not been out long enough’ ***Respondent 121***  ‘Not had vaccine in pregnancy’ ***Respondent 135***  ‘I haven’t had it.’ ***Respondent 144***  ‘I have not had the vaccine while pregnant. I will not be having it until I have given birth.  ‘I did not have it during pregnancy as it’s so new I was worried about future issues for my unborn child’ ***Respondent 50***  ‘Wouldn’t have it pregnant or after pregnancy’ ***Respondent 255*** |
| **Prefer to wait until later in pregnancy/ after pregnancy** |
| ‘I think I would have waited until the 2nd or even 3rd trimester’ ***Respondent 1***  ‘I probably wouldn’t because it’s so new and I would isolate and have it later, which is pretty much what did happen with me. Purely to err on the safe side’ ***Respondent 40***  ‘I’m not having it until after I’ve had my baby’ ***Respondent 58***  ‘Decided against the vaccine whilst pregnant. Will have it after pregnancy. Too new / not enough research on its effects’ ***Respondent 155***  ‘Opted to wait for my booster until I have had my baby’ ***Respondent 64***  ‘I chose to wait and have my vaccination following the birth’ ***Respondent 88***  ‘I waited to have my second until I was further along in my pregnancy as I was scared it would the effect my baby’ ***Respondent 105***  ‘I delayed my vaccination until after the first trimester.’ ***Respondent 124***  ‘I won't be having the booster until after pregnancy’ ***Respondent 126***  ‘I have decided to wait until my third trimester to get vaccine as we had no data on getting vaccine in early pregnancy’ ***Respondent 129***  ‘No, I’d rather wait till after my pregnancy’ ***Respondent 154***  ‘Unvaccinated, waiting till after birth’ ***Respondent 167***  ‘No I have not had my COVID vaccinations during my pregnancy. I will However have them once my baby is born and I know we are both healthy’ ***Respondent 172***  ‘I have not had the vaccine while pregnant. I will not be having it until I have given birth.’ ***Respondent 184***  ‘Had 2 doses before pregnancy, waiting until end of pregnancy for booster due to concerns of future effects on baby’ ***Respondent 230*** |
